# Primary care supply and quality of care in England

**DOI:** 10.1007/s10198-017-0898-2

**Published:** 2017-05-30

**Authors:** Laura Vallejo-Torres, Stephen Morris

**Affiliations:** Department of Applied Health Research, University College London, Gower Street, London, WC1E 6BT UK

**Keywords:** General practitioner, Health care supply, Quality of care, Multilevel model, Primary care, I110, I180

## Abstract

We investigated the relationship between primary care supply and quality of care in England. We analysed 35 process measures of quality of care covering 13 medical conditions using English Longitudinal Study of Aging data linked to area of residence indicators. Greater GP density had a statistically significant and positive association with quality of care, and distance to GP practice had a statistically significant and negative association. The effects were concentrated in indicators of care related to cardiovascular diseases and arthritis, and on specific indicators for diabetes, incontinence and hearing problems. The results suggest that better primary care supply can improve quality of care.

## Introduction

In England, as in many countries, primary care plays an important role in managing the health of the population. Individuals register with general practices that provide a range of primary care services involving the diagnosis of ill health, referral to secondary care services, prescribing, direct management of acute illness and long-term conditions, and health promotion [1]. These services are coming under mounting pressure due to: higher demand caused by an ageing population and larger numbers of patients with comorbidities; tighter budgetary constraints; and the widening role of primary care to meet the health care needs of the population, in terms of a shift from hospital-based to community-based care and a move towards general-practice-led commissioning [2].

In England in 2014 there were 37,000 full-time equivalent (FTE) general practitioners (GPs), 15,000 FTE general practice nurses, and 73,000 FTE other practice staff working in under 8000 general practices in the National Health Service (NHS), with each practice serving a mean population of 7,000 patients [3]. While sizable, it has been argued that the primary care workforce has insufficient capacity to meet the demands placed on it [4], leading to concerns about the quality of care [5].

The aim of this study was to investigate the relationship between the supply of primary care and the quality of that care (QoC). Our hypothesis was that primary care supply has a positive impact on QoC. We expected that increasing primary care supply should improve access to primary care for patients, and increase the number and length of primary care contacts. Increased contacts with patients ought to improve QoC because GPs can better adhere to appropriate standards of care, communicate better with their patients and improve diagnosis, and can broaden the range of services they provide to patients. Also, in the NHS where patients can switch GPs and health care is free at the point of receipt, GPs are expected to compete for patients on the basis of non-price factors such as QoC. However, it may be that increasing primary care supply has no impact on QoC, because GPs are not perfect agents for patients [6].

QoC can be evaluated using structural measures, process measures, or outcomes [7]. Structural data are characteristics of the health care system; process measures describe what is being done to patients; outcomes refer to patient subsequent health status. Primary care supply is a structural measure, and so these are not suitable QoC measures in our study. It has been argued that outcomes are not appropriate measures of QoC in primary care because they depend on all levels of health care (primary, secondary, and tertiary) and because they depend on factors unrelated to health care such as socioeconomic status. Process measures are generally accepted as the most useful indicators of QoC in primary care [8] and we focus on those here. We use 35 individual level process measures of QoC covering 13 medical conditions, which were derived to assess the care received by older people. Self-reported data on these measures were available at the individual level, and collected at repeated points in time over several years, for participants in the English Longitudinal Study of Ageing (ELSA) [9]. They have also been used in other studies to measure QoC [10–12], though none of these has evaluated the impact of primary care supply.

## Previous research

The relationship between primary care supply and QoC has been investigated in other countries [13–15] with some studies showing a statistically significant and positive association and some showing a non-significant association. To our knowledge, the present study is the first English study.

Evidence from several studies suggests that greater primary care supply, usually measured in terms of the number of GPs per capita, is positively correlated with better health outcomes [16, 17]. The relationship holds at different units of analysis (countries, areas within countries, individuals) for various health outcomes including all-cause mortality [17–26], cause-specific mortality [17, 19–21, 23–25, 27–29], teenage conception rates [23, 24], early cancer detection [30–32], self-reported health [24, 33–37], obesity [38], and health inequalities [16, 39]. Many of the studies were undertaken at the ecological level, and few accounted for endogeneity of primary care supply [36].

There have been few studies analysing the relationship between primary care supply and process measures of QoC. Perrin and Valvona [13] examined the impact of physician density on quality of care in the USA, measured in terms of appropriate, discretionary and inappropriate ordering of ancillary tests. There was some evidence that discretionary testing increased with physician supply, and that testing of all types was negatively associated with physician supply. The effects were small and it was unclear if they were significantly different from zero; appropriate, discretionary and inappropriate testing were not clearly defined; the test data were linked to physician density data measured 5–7 years afterwards; there were no controls for confounding factors. Besides that, the institutional and organisational environment of primary care in the USA is different from that in England.

Rizzo and Zeckhauser [14] examined the impact of physician advertising on the price, quality and quantity of physician services using USA physician survey data for 1987–1988. As part of their study they measured the impact of physician supply (predicted natural logarithm of the number of physicians per capita in the country where the physician resides in 1986) on quality (mean physician time spent per patient visit). Physician supply was potentially endogenous and so was instrumented based on the percentage of the labour force who were white-collar workers, population size and rate of change, local house values and crime rates. The results showed a non-significant impact of physician supply on mean physician time spent per patient office visit, and a positive impact on mean physician time spent per patient visit in all practice settings that was statistically significant at the 10% level but not at the 5% level. Estimates that did not control for endogeneity were very similar.

More recently, Jurges and Pohl [15] used German data for 2004 from the Survey of Health, Aging, and Retirement in Europe to study the relationship between GP supply (number of GPs per 100,000 residents, number of GPs per 100,000 residents aged 50 and over, number of GPs per 100 square kilometres) and QoC provided to older adults. QoC was measured as the degree of adherence to medical guidelines for the management of risk factors for cardiovascular disease (CVD) and prevention of falls reported by patients. The outcome variable was the percentage of recommended care received by respondents, based on the percentage of three care guidelines for CVD and two care guidelines for falls that were met. Patient-level separate QoC variables for CVD risk and falls were regressed against GP supply plus individual and area covariates. The associations between GP supply and QoC were statistically non-significant. This result remained after a series of robustness checks testing non-linear functional forms and controlling for endogeneity.

In the present study, we build on Jurges and Pohl’s (2012) approach, using a wider range of QoC indicators that cover more medical conditions, some of which are available for the same individuals over multiple years. Differently to Jurges and Pohl, we run analyses at the indicator level, controlling for the complex multilevel nature of our data, and measure primary care supply using measures of GP density and distance to practice.

## Economic framework

**Fig. 1:**
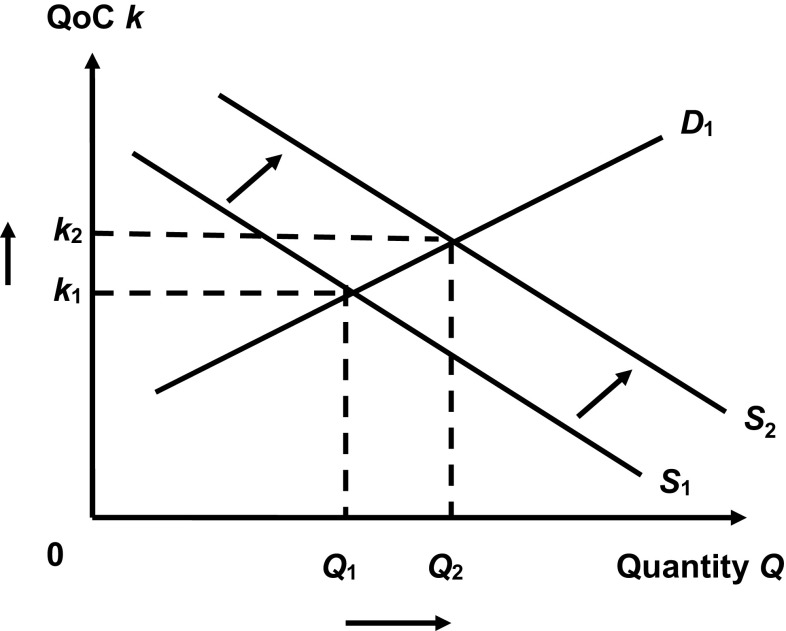
Relationship between demand, supply and quality of care

We expect a positive association between primary care supply and QoC for two reasons. First, we expect that higher levels of primary care supply are associated with better access to primary care services for patients, for example in terms of higher numbers and longer lengths of primary care contacts. Better access ought to improve QoC because GPs can better adhere to appropriate standards of care and can broaden the range of services they provide to patients. Second, higher levels of GP supply entail more competition among GPs for patients. If patients value QoC and if payments are zero at the point of receipt of care (as is the case in the UK), then GPs compete on the basis of non-price factors, e.g. by increasing QoC. Therefore, all else equal, QoC should be positively correlated with GP supply. This is shown in Fig. 1. Demand for GP services *Q*_*D*_ increases with QoC *k*, shown by curve *D*_1_. Better QoC increases the marginal cost of providing additional units of primary care, so supply *Q*_*S*_ decreases with quality, shown by curve *S*_1_. *S*_1_ and *D*_1_ are not supply and demand curves in the usual sense because the *y*-axis depicts quality not price, *S*_1_ is downward sloping from left to right, and *D*_1_ is upward sloping from left to right. The equilibrium quality and quantity in this model are *k*_1_ and *Q*_1_, respectively. An exogenous increase in GP supply shifts the supply curve outwards to *S*_2_. At the new equilibrium, *k*_2_ and *Q*_2_, both quality and quantity are higher than at the initial levels. Hence, in this model increases in supply lead to increases in quality.

Jurges and Pohl [15] provide a formal illustration of this model, which is directly applicable to our analysis and so we use it to illustrate our model, acknowledging that we draw heavily on their work. They construct a model whereby GPs are price-takers and set quality *k* to maximise profit, which is a realistic scenario in the NHS in England. Patients receive benefits *b* from treatment, which are a function of quality *b* = *b*(*k*), and GPs incur costs *c* per patient to provide treatment; costs are also a function of quality *c* = *c*(*k*). Assume that patient benefits increase with quality but at a decreasing rate ($\partial b/\partial k = b^{\prime} > 0,\;b^{\prime\prime} < 0$) and costs increase with quality at an increasing rate ($\partial c/\partial k = c^{\prime} > 0,\;c^{\prime\prime} > 0$). The number *n* of patients on each GP’s list is a function of the benefits provided by that GP and GP supply *s* where *s* is increasing in GP density and decreasing in distance to GPs’ practice, *n* = *n*(*b*(*q*), *s*). GP list size is assumed to increase with patient benefits but at a decreasing rate ($\partial n/\partial b > 0,\;\partial^{2} n/\partial b^{2} < 0$) and to decrease with GP supply ($\partial n/\partial s < 0$), and we assume there is no interaction between patient benefits and GP supply in terms of how they affect list size ($\partial^{2} n/\partial b\partial s = 0$).

GP income comprises two elements. The first is capitation income, which is a function of list size *n* and capitation payments per patient *p*. The second is quality-based income awarded as part of the Quality and Outcomes Framework (QOF) to GPs based on the average quality of services provided to all patients. This is derived from the proportion of patients on the GP’s list treated in accordance with pre-defined quality criteria for which GPs are awarded points *t*. Points are a function of quality *t* = *t*(*k*), and increase with quality but at a decreasing rate ($\partial t/\partial k = t^{\prime} > 0,\;t^{\prime\prime} < 0$). GPs are paid a fixed amount *r* per point achieved. Quality-based income is assumed to be independent of list size. Since GPs are price-takers, *p* and *r* are set exogenously. GP income *y* is given by:

1 $$y = n(b(k),s)p + rt(k),$$

and profit *π* is given by:

2 $$\pi = n(b(k),s)(p - c(k)) + rt(k).$$

GPs are assumed to maximise profit subject to two constraints. The first is to break even with each patient treated:

3 $$p - c(k) \ge 0,$$

and the second is that patient benefits must exceed some minimum level *b* ^min^:

4 $$b(k) - b^{\hbox{min} } \ge 0.$$

Following Jurges and Pohl (2012), if we assume an interior solution then optimal quality changes as primary care supply changes according to the following expression:

5 $$\frac{\partial k}{\partial s} = \frac{{\frac{\partial n}{\partial s}c^{\prime}}}{{\left( {\frac{{\partial^{2} n}}{{\partial b^{2} }}b^{\prime 2} + \frac{\partial n}{\partial b}b^{\prime\prime}} \right)\left( {p - c\left( k \right)} \right) - 2\frac{\partial n}{\partial b}b^{\prime}c^{\prime} - nc^{\prime\prime} + rt^{\prime\prime}}}.$$

This is identical to the result in Jurges and Pohl [15], except for the last term in the denominator on the right hand side of Eq. [5], which accounts for the impact of quality-based income provided under the Quality and Outcome Framework in the UK. Given the assumptions made above then the numerator and the denominator of Eq. [5] are both less than zero and $\partial k/\partial s > 0.$

## Data and variables

Our analysis is based on data from the English Longitudinal Study of Aging (ELSA), which provides data from a representative sample of adults aged 50 or more living in private households in England. The sample was drawn from households that participated in the Health Survey for England (HSE) in 1998, 1999 or 2001. Individuals selected for the ELSA survey have been interviewed every 2 years since 2002.

We use data from waves 2, 3 and 4 (surveyed in 2004–2005, 2006–2007 and 2008–2009, respectively) of ELSA, which provide detailed information on the quality of health care received as well as measures of health status, demographic and socioeconomic factors, and for which we were able to obtain Primary Care Trust (PCT) codes of residence under special license from the data owners. PCTs were responsible for commissioning health care services during the data period of analysis. Clinical Commissioning Groups (CCGs) gradually replaced PCTs which were finally abolished in April 2013. England was divided into 303 PCTs during wave 2 and 152 PCTs during waves 3 and 4. We use the PCT Mapping Tool^1^ to convert data for the 303 PCT structure to the 152 PCT structure and use area-based measures for the 152 PCT structure. The number of individuals included in our data is 8676; most participated in more than one wave, yielding a total of 21,571 observations.

### Quality of care indicators

We analyse 35 indicators of quality of primary care covering 13 medical conditions. The indicators are defined in Table 6 in the Appendix. Each definition comprises an eligibility statement describing the patients in whom the indicator ought to be applied (e.g. “If aged 50 or over and has diabetes”), and a question to ascertain whether or not the QoC standard has been met (e.g. “In the past year, has any doctor or nurse examined your bare feet?”). The response to the question is binary (yes/no), where a yes response means that the quality of care standard has been met. The indicators were derived to assess the care of vulnerable older people across a number of conditions [11]. The conditions were chosen according to their prevalence, impact, effectiveness of available prevention/treatment, importance in older people, feasibility of measurement, and the potential for quality improvement. The indicators were designed to represent processes of care that have been linked to improved outcomes in each of these conditions, and were constructed with input from an expert panel of clinicians, who were asked to review and score the degree to which the indicators reflected good practice in the UK. All indicators were intended to assess the quality of the delivery of care to a minimum acceptable standard, rather than the optimal level [11], and are based on individual self-reporting by patients.

### Supply of primary care indicators

We use two measures of primary care supply: GP density (number of GPs per 1000 patients), and GP distance (the average distance to a general practice).

The GP density variable was defined as the mean number of full-time equivalent (FTE) GPs per 1000 registered patients in the PCT of residence of the individual. Information on the number of GPs was taken from the NHS staff numbers database available from the NHS Information Centre^2^ Data on the number of GPs in 2006 was linked to waves 2 and 3 of ELSA and data for 2008 was linked to wave 4 of ELSA. Data on NHS staff numbers prior to 2006 was reported using the 303 PCT structure and is not used in our analysis because the PCT Mapping Tool is not considered suitable for staff data.^3^ In a supplementary analysis we used a different linkage to introduce more variation in the GP density variable, mapping data on the number of GPs from 2006, 2007 and 2009 to waves 2, 3 and 4, respectively (see robustness checks below).

The GP distance variable was taken from the Barriers to Housing and Services domain of the Index of Multiple Deprivation (IMD) 2004 and 2007 extracted from the Neighbourhood Statistics website.^4^ It measures the mean road distance in kilometres measured from each population weighted Census Output Area centroid to the nearest GP premises. GP premises locations were used rather than GP practice locations, as GP practices may be administrative addresses only and not where GPs actually see patients, and practices may have multiple premises. The data were available at the Lower Super Output Area (LSOA) level (of which there are 32,482 in England) and aggregated to PCT level by calculating the weighted mean value across all LSOAs within each PCT, weighted by the proportion of the PCT population living in each LSOA. Distance to GP premises data from the IMD 2004 was applied to wave 2 of ELSA, while the IMD 2007 data was linked to waves 3 and 4.

As a robustness check, we explored the potential non-linear effect of primary care supply on quality of care by using different functional forms of the GP density and GP distance variables (see below).

### Other covariates

A comprehensive set of individual and area covariates were included in our models. The individual covariates were age, gender, marital status (4 categories), ethnic group (2 categories), self-reported health status (2 categories), educational attainment (7 categories), employment status (5 categories) and total net household financial wealth. The financial wealth variable was derived in ELSA using very detailed data on a number of financial elements such as savings and debts. The area covariates were the age profile of the population (5 categories) and the percentage of individuals belonging to the white ethnic group based on data extracted from the Office of National Statistics. We also included a measure of area deprivation — the percentage of individuals aged 25–54 in the area with no or low qualifications. This was extracted from the Education, Skills and Training domain of the IMD 2004 and 2007 and linked to individual data using PCT codes using the same process described above. This variable was used as it provides a measure of deprivation that can be compared across both versions of the IMD, allowing more variation in the variable across time, as opposed to IMD variables reported as scores which cannot be meaningfully combined across IMD 2004 and 2007. Our chosen measure is highly correlated with other area deprivation measures. We experimented with including a wider set of area covariates in the robustness checks related to IMD 2004 scores only. These were also used as instruments for GP supply in an instrumental variable (IV) specification that we ran as a robustness check (see below).

## Econometric approach

We explore the impact of primary care supply on QoC using regression analysis. We first analyse the impact of primary care supply on all the QoC indicators combined, running analyses at the indicator level adjusting for different clustering structures. We then construct models combining QoC indicators by disease area as well as investigating each of the 35 indicators separately. Our data allow us to explore different specifications accounting for multiple responses for each individual (related to different indicators), as well as repeated observations measured over time.

### Pooled analyses

The full list of the 35 QoC indicators was only included in wave 2. We therefore ran an analysis combining information on the 35 indicators using data only from this wave.

In ELSA, individuals only responded to the QoC indicators that were relevant to them, based on the eligibility statements (Table 6 in Appendix). Data on all of the 35 indicators were not available for every individual, and some people might have responded to multiple indicators. We therefore created a dataset with observations for every indicator by individual. This dataset could potentially have 303,660 observations (8676 individuals in wave 2 times 35 QoC indicators), but not all people in wave 2 met the eligibility statements, and the actual number of observations was 23,659.

To account for the hierarchical structure of our data we estimated multilevel regression models that explicitly account for the fact that observations are nested within groups (QoC indicator level responses are nested within individuals, individuals are nested within PCTs). Multilevel models have also been recommended when multiple measures of an outcome, in our case quality of care indicators, are available [40]. The 3-level model is:

6 $$k_{mij} = \alpha^{'} + \beta^{'} s_{j} + \delta^{'} Z_{ij} + \mu_{i} + u_{j} + \varepsilon_{mij} ,$$

where *k* denotes QoC associated with indicator *m* for individual *i* living in PCT *j*. *s* is primary care supply, *Z* are other indicators included in the regression model such as demographic, socioeconomic indicators and area deprivation, and *ε* is an error term. *s* is available at the area (PCT) level only, while *Z* includes individual level covariates and area level indicators. *β* is the coefficient of interest to be estimated. The QoC indicators *k* are binary variables taking the value 1 if the indicator is met, and 0 otherwise. For all models we use logistic regression and report odds ratios. *μ*_*i*_ and *u*_*j*_ are the individual-specific and PCT-specific error components, respectively, capturing unobserved heterogeneity at these levels, and the other variables are defined as before. It is assumed that *μ*_*i*_ and *u*_*j*_ have zero mean and unknown variances to be estimated, and they are independent of *ɛ*_*mij*_ and the regressors of the model. To examine the importance of the different clustering effects we also ran 2-level models, modelling indicators nested within individuals and indicators nested within PCTs.

### Analyses by disease area

We derive subgroups of indicators based on the disease area. We consider 13 different conditions. The number of indicators included in each disease area varied from 1 to 7 indicators (see Table 1 and Table 6 in Appendix).

**Table 1:** Summary statistics of quality of care indicators by disease area

Disease area	Number of QoC indicators	Mean	SD	ELSA wave	Sample
All	35	0.633	0.482	2	23,681
Hypertension	3	0.772	0.420	2–4	10,514
High cholesterol	2	0.636	0.481	2	3311
IHD	3	0.729	0.445	2	1841
Stroke	1	0.417	0.494	2–4	263
Diabetes	7	0.587	0.492	2–4	10,892
Osteoarthritis	5	0.350	0.477	2–4	7430
Osteoporosis	2	0.669	0.471	2	924
Prevention of falls	2	0.303	0.459	2 and 4	1918
Pain	1	0.771	0.421	2 and 4	462
Incontinence	4	0.519	0.500	2	2089
Vision	1	0.571	0.495	2	591
Hearing	2	0.790	0.407	2	2318
Depression	2	0.637	0.481	2 & 4	786

ELSA waves 2, 3 and 4 were in 2004–2005, 2006–2007 and 2008-2009, respectively. All QoC indicators are coded so that 1 = indicator is met, 0 otherwise

*QoC* quality of care, *IHD* ischaemic heart disease

Information on the QoC indicators for some conditions were collected in wave 2 only. These include indicators for high cholesterol, ischaemic heart disease (IHD), osteoporosis, incontinence, and vision and hearing problems. The econometric approach to analysing these sets of indicators is equivalent to the one described in the previous section as there are not repeated observations over time.

For the remaining disease-specific groups of indicators (hypertension, stroke, diabetes, osteoarthritis, falls, pain and depression) information was collected in more than one wave. In these cases we make use of the repeated observations over time for each individual. For indicators *m* nested within individual *i*, responding in time period *t*, and living in PCT *j*, the model takes the form:

7 $$k_{mitj} = \alpha^{\prime} + \beta^{\prime}s_{tj} + \delta^{\prime}Z_{itj} + t + \mu_{i} + u_{j} + \varepsilon_{mitj} .$$

Repeated responses over time are nested within individuals, therefore the models continue to be structured in a 3-level multilevel model. We add year indicators *t* to control for year fixed effects.

### Analyses of individual indicators

We analyse the impact of primary care supply on each of the 35 indicators of QoC separately. For those disease areas where only one indicator of QoC was available (stroke, vision and pain) these analyses are equivalent to those undertaken in the previous section.

The model to investigate the impact of primary care on individual indicators collected in only one wave is defined as a 2-level multilevel model for individual *i* living in PCTs *j*:

8 $$k_{ij} = \alpha^{\prime} + \beta^{\prime}s_{j} + \delta^{\prime}Z_{ij} + u_{j} + \varepsilon_{ij} .$$

For individual indicators collected in more than one wave we have repeated observations within individuals, and we use a 3-level model with *i* individuals responding in *t* time periods and living in *j* PCTs:

9 $$k_{tij} = \alpha^{\prime} + \beta^{\prime}s_{tj} + \delta^{\prime}Z_{itj} + t + \mu_{i} + u_{j} + \varepsilon_{tij} .$$

## Predicting PCTs allocation of GP workforce to achieve QoC targets

We used the results of the model that includes information on the 35 QoC indicators (Eq. [6]) to predict the optimal allocation of GP supply across the 152 PCTs that would be required to achieve specific targets for QoC. We based these targets on the thresholds used by the Quality and Outcomes Framework (QOF) to define achievement in quality of care provided by GP practices. The objective of the QOF is to improve QoC by rewarding practices for the quality of care they provide to their patients, across four domains of care (clinical, organisational, patient experience, additional services), and 148 achievement measures. At the time when the data was collected, achievement was defined using maximum thresholds of between 70 and 90% for most measures, with few exceptions [41]. We used both 70 and 90% as potential targets for QoC. The analysis does not show the number of GPs needed to meet QoF targets, but the number needed to obtain QoC scores of 0.7 and 0.9 from our analysis.

## Results

### Summary statistics

Summary statistics for all the QoC indicators combined and by disease area are in Table 1. Summary statistics for each of the 35 indicators are in Table 5 alongside the main results for each separate indicator. The probability that QoC standards were met across all 35 indicators combined in wave 2 in ELSA was 63.3%. For the individual indicators, the probability that care standards were met ranged from 30.3 to 79%. Standards of care for hypertension, IHD, pain and hearing problems are among those more likely to be met (all > 70%), while quality of care standards for prevention of falls, osteoarthritis and stroke are the least likely to be achieved (all < 50%).

Table 2 summarises the characteristics of the study population at wave 2. On average there were 0.59 GPs per 1000 registered patients, and the mean distance to GP premises was 1.6 km. The mean age of the sample was 66 years and 55% were females. The majority of the sample were married or widowed, 98% were white and 28% reported bad or very bad health. Over 38% of the sample had no qualifications and nearly 53% were retired. The mean net household financial wealth was £57,827. On average, the percentage of adults with no or low qualifications in the area of residence of the individual was 43%, the percentage of individuals aged 65 and over was nearly 20%, and 91% of the area population belonged to the white ethnic group.

**Table 2:** Summary statistics of covariates (ELSA wave 2)

	Mean	SD
GP supply		
GPs per 1000 patients (number)	0.589	0.075
Distance to practice (km)	1.585	0.674
Demographics		
Age (years)	66.487	9.764
Female	0.552	0.497
Marital status		
Married	0.657	0.475
Single	0.051	0.221
Divorced	0.107	0.309
Widowed	0.184	0.388
Ethnic group		
White	0.977	0.151
Self-reported health		
Bad health	0.282	0.450
Education		
Degree	0.123	0.329
Higher (less than degree)	0.120	0.325
A levels	0.067	0.250
GCSE	0.170	0.376
CSE	0.047	0.211
Other qualifications	0.089	0.285
No qualifications	0.383	0.486
Employment status		
Employed	0.297	0.457
Retired	0.531	0.499
Unemployed	0.008	0.087
Permanently sick	0.055	0.228
Family carer	0.102	0.303
Net household financial wealth indicator (£)	57,827	20,246
Area level characteristics		
Percentage with no qualifications in area (%)	43.021	6.653
Population aged 0–15 (proportion)	0.173	0.035
Population aged 16–29 (proportion)	0.220	0.021
Population aged 30–44 (proportion)	0.222	0.022
Population aged 45–64 (proportion)	0.192	0.032
Population aged 65 and over (proportion)	0.173	0.035
Percentage white ethnic group (%)	91.158	9.888
Sample	8876	

Unless otherwise indicated, all variables are binary variables taking the value 1 if the respondent is in that category and 0 otherwise

### Pooled analyses

The results combining the 35 QoC indicators in wave 2 are reported in Table 3. The main finding is that primary care supply and QoC are positively correlated. Quality of care standards were significantly more likely to be achieved if the individual lived in an area with higher GP density and shorter distance to practice. The confidence interval around the odds ratios for these variables becomes wider when we account for the full hierarchical structure of the data in the 3-level model, but the odds ratios were significantly different from one at the 5% level. We computed marginal effects for the fixed part of this model, and estimated that an increase in one GP per 1000 patients increases the probability of meeting QoC standards by approximately 20%, while an increase in 1 km in the average distance to GP premises decreases the probability of meeting QoC standards by 2%.

**Table 3:** Logit models for the probability of meeting quality of care indicators — all indicators combined (ELSA wave 2)

	2-level model		2-level model		3-level model	
Level 1:	Indicators		Indicators		Indicators	
Level 2:	PCTs		Individuals		Individuals	
Level 3:					PCTs	
	OR	[95% CI]	OR	[95% CI]	OR	[95% CI]
GPs per 1000 patients	2.130**	[1.067–4.254]	2.657***	[1.428–4.943]	2.453**	[1.137–5.291]
Distance to practice (km)	0.927**	[0.862–0.997]	0.916**	[0.851–0.986]	0.918**	[0.843–1.000]
Age	0.996*	[0.992–1.000]	0.997	[0.991–1.002]	0.997	[0.992–1.002]
Female	0.866***	[0.816–0.919]	0.859***	[0.793–0.930]	0.859***	[0.793–0.929]
Single	0.867**	[0.761–0.988]	0.818**	[0.688–0.972]	0.818**	[0.689–0.972]
Divorced	1.003***	[0.916–1.097]	0.994	[0.882–1.121]	0.991	[0.879–1.117]
Widowed	0.891	[0.827–0.961]	0.868***	[0.785–0.960]	0.865***	[0.782–0.956]
White	0.922	[0.782–1.086]	0.944	[0.754–1.182]	0.941	[0.751–1.179]
Bad health	1.048	[0.989–1.111]	1.055	[0.976–1.141]	1.059	[0.979–1.145]
Higher (less than degree)	1.025	[0.906–1.159]	1.035	[0.880–1.216]	1.031	[0.877–1.211]
A level	0.928	[0.803–1.071]	0.912	[0.754–1.103]	0.905	[0.749–1.095]
GCSE	0.912	[0.815–1.022]	0.885	[0.762–1.027]	0.888	[0.765–1.030]
CSE	0.941	[0.808–1.097]	0.942	[0.769–1.154]	0.945	[0.772–1.156]
Other qualifications	0.983	[0.865–1.116]	0.953	[0.804–1.129]	0.954	[0.806–1.130]
No qualifications	0.932	[0.842–1.031]	0.930	[0.813–1.064]	0.927	[0.810–1.060]
Retired	1.027	[0.940–1.121]	1.037	[0.925–1.164]	1.039	[0.926–1.165]
Unemployed	1.147	[0.835–1.576]	1.270	[0.832–1.937]	1.264	[0.830–1.926]
Permanently sick	1.032	[0.921–1.157]	1.065	[0.913–1.243]	1.060	[0.909–1.237]
Family carer	1.106*	[0.983–1.245]	1.144*	[0.980–1.335]	1.143*	[0.979–1.334]
Financial wealth	1.000	[1.000–1.000]	1.000	[1.000–1.000]	1.000	[1.000–1.000]
%with no qualification in area	0.994	[0.986–1.002]	0.994	[0.987–1.002]	0.994	[0.984–1.003]
Proportion aged 0–15	2.889	[0.054–153.6]	1.695	[0.036–79.869]	2.407	[0.025–228.7]
Proportion aged 30–44	0.111	[0.002–5.227]	0.154	[0.003–7.318]	0.139	[0.002–12.05]
Proportion aged 45–64	0.151	[0.003–9.007]	0.069	[0.001–3.782]	0.089	[0.001–9.810]
Proportion aged 65+	0.554	[0.022–14.01]	0.558	[0.026–12.078]	0.658	[0.017–25.51]
%White ethnic group	1.008**	[1.001–1.015]	1.008**	[1.001–1.015]	1.008*	[0.999–1.016]
*N*	23,659		23,659		23,659	

*PCT* primary care trust, *OR*  odds ratio, *CI* confidence interval, *GP* general practitioner

* *P* < 0.1, ** *P* < 0.05, *** *P* < 0.01

Looking at the other covariates, we observe that females are significantly less likely to receive care that meets the minimum acceptable standards compared with males. Compared with being married, those who are single or widowed are less likely to meet the quality of care indicators. Family carers are more likely to achieve the indicators of quality of care compared with those who are employed, and those living in an area with a higher percentage of white population are significantly more likely to receive care that meets the quality standards.

### Analyses by disease area

The results using the 3-level multilevel modelling approach for each of the disease domains are presented in Table 4. We consider 13 conditions separately, and in addition we combine the indicators of hypertension, high cholesterol, stroke, IHD and diabetes into a cardiovascular disease (CVD) group.

**Table 4:** Logit models for the probability of meeting quality of care indicator — by disease area (all numbers are odds ratios)

	CVD^a^	Hypertension^b^	High cholesterol^a^	IHD^a^	Stroke^b^	Diabetes^b^	Osteoarthritis^b^
GPs per 1000 patients	1.643	1.166	7.723*	1.228	16.14	1.207	4.976**
Distance to practice	0.915*	0.875*	0.862	0.913	0.809	0.926	0.909
Age	0.990***	1.030***	0.979***	0.979**	0.978	0.995	0.994
Female	0.942	0.767***	1.001	0.805	1.158	0.958	1.073
Single	0.901	0.821	0.706	0.597*	1.030	0.740***	0.896
Divorced	1.016	1.005	0.916	0.982	0.726	0.845	0.844
Widowed	0.857**	0.938	0.777*	0.758	0.763	0.805***	0.940
White	1.008	0.587**	0.733	0.963	2.678	0.984	0.568**
Bad health	1.061	1.089	0.911	2.122***	0.770	1.040	1.256***
Higher (less than degree)	1.039	1.311*	0.711	1.504	3.687	1.095	0.835
A level	0.964	1.205	0.736	1.114	3.555	1.044	0.859
GCSE	0.935	1.241	0.648**	1.103	0.669	1.002	0.738**
CSE	0.949	1.118	0.574*	1.101	1.603	0.958	0.839
Other qualifications	0.956	1.200	0.677	0.872	4.155	1.098	0.739*
No qualifications	0.853*	1.078	0.603**	1.023	1.256	0.858*	0.827
Retired	1.113	1.095	1.013	1.824***	1.668	1.113	1.148
Unemployed	1.257	0.949	1.226	1.464	–	1.405	1.341
Permanently sick	0.993	1.246	0.847	1.457	1.141	0.954	1.633***
Family carer	1.159	1.125	1.215	1.563*	2.379	1.159	1.045
Financial wealth	1.001*	1.000	1.002***	0.999	0.995	1.000	1.000
% with no qualification in area	0.995	1.001	1.001	0.983	1.044	0.998	0.999
Proportion aged 65+	4.504	3.340	7.862	0.057	0.095	1.563	4.924
% White ethnic group	1.003**	1.009	0.993	1.007	0.999	1.008**	0.997
Wave 3	–	3.836***	–	–	0.411	0.937	0.846**
Wave 4	–	5.819***	–	–	0.632	0.905*	0.853*
*N*	13,999	10,514	3311	1841	263	10,892	7430

	Osteoporosis^a^	Falls^c^	Pain^c^	Incontinence^a^	Hearing^a^	Vision^a^	Depression^c^
GPs per 1000 patients	1.481	3.090	4.992	3.665	8.227	0.950	1.083
Distance to practice	1.013	1.323	0.946	0.852	1.099	0.963	0.896
Age	1.014	1.008	1.017	0.992	1.054***	1.031**	0.987
Female	0.980	1.264	1.103	0.972	1.174	1.355	0.894
Single	0.491*	1.862	0.568	0.652	0.241***	1.155	0.936
Divorced	1.100	1.148	1.430	1.072	0.823	1.642	1.248
Widowed	0.762	0.941	0.487**	1.054	0.505***	1.342	1.157
White	0.586	0.580	0.947	0.693	0.856	0.484	2.811*
Bad health	0.946	3.094***	0.587**	1.106	1.516**	1.564**	1.142
Higher (less than degree)	0.849	1.487	0.905	1.104	0.875	0.590	0.697
A level	0.978	1.591	1.210	0.691	0.519	0.386	0.431**
GCSE	0.538	1.339	0.453	0.756	0.564	0.910	0.715
CSE	1.747	1.456	0.532	0.809	0.412*	0.578	1.291
Other qualifications	1.184	1.291	0.570	0.973	0.635	0.623	0.412**
No qualifications	0.768	1.235	0.403*	1.072	0.709	0.740	0.595*
Retired	1.155	2.336**	1.365	1.092	0.863	0.502	0.905
Unemployed	0.551	0.592	–	3.246	0.395	–	1.147
Permanently sick	1.535	2.800**	1.038	1.158	1.142	0.659	1.386
Family carer	1.067	1.124	4.489**	1.102	0.724	0.562	1.161
Financial wealth	1.001	0.999*	0.999	0.999	1.001	0.999	1.000
% with no qualification in area	1.000	0.991	1.033*	0.993	1.011	0.973*	1.006
Proportion aged 65+	0.006	0.000***	21.858	0.001**	184.6	0.002	538.03
% White ethnic group	1.014	1.037**	0.990	1.030***	0.987	1.004	0.968**
Wave 3	–	1.000	–		–	–	1.000
Wave 4	–	0.565***	0.820		–	–	0.826
*N*	924	1918	462	2089	2318	591	786

All models are based on a 3-level multilevel regression — indicators & individuals & PCTs

* *P* < 0.1, ** *P* < 0.05, *** *P* < 0.01. CVD = Cardiovascular diseases; combines indicators for hypertension, high cholesterol, IHD, stroke and diabetes

^a^Data from wave 2 in ELSA

^b^Data from wave 2 to 4 in ELSA

^c^Data from wave 2 & 4 in ELSA

We find evidence of a positive effect of a larger number of GPs in the area on quality of care indicators for high cholesterol and arthritis. In most of the remaining disease domains the number of GPs per 1000 patients have a positive effect (odds ratios >1) but the effect is not statistically significant. The impact of the average distance to GP premises is negative and statistically significant on CVD QoC indicators and hypertension, and it is generally negative in the remaining disease specific models, but not statistically significant.

The comparison of the effect of the other covariates across disease domains shows some interesting results. The impact of age on QoC varies by disease, with a positive effect in hypertension, hearing and vision problem indicators, and a negative impact for indicators related to high cholesterol and IHD. Females are significantly less likely to receive care that meets hypertension standards, while being single or widowed has a negative impact on QoC for a number of disease areas. Non-white ethnic groups are more likely to meet standards of care for hypertension, arthritis and depression, and those reporting bad health have generally a larger probability of meeting care standards, with the exception of pain management. Individuals with educational attainment lower than a degree are generally less likely to meet the minimum standards of care, especially for high cholesterol, arthritis and depression. Being permanently sick, retired or taking care of the family increases the probability for some disease-specific indicators as compared with those who are employed. Larger net financial wealth increases the probability of CVD and high cholesterol good management, but reduces the likelihood of meeting prevention of falls standards of care. Area deprivation decreases the probability of achieving vision problems QoC indicators, but increases the probability of good pain management. The proportion of individuals aged 65 and over residing in the area where the individual lives (the remaining age categories were dropped due to small samples) has a significant and negative effect on meeting QoC standards for falls and incontinence problems. Finally, while the probability of meeting standards of care for hypertension has increased over time, the probability of achievement of QoC indicators for arthritis, diabetes and falls was lower in 2008–2009 compared to 2004–2005.

### Analyses of individual indicators

The estimates of the impact of the area supply of primary care measures in each of the 35 indicators of quality of care separately are presented in Table 5. All the models control for the same covariates as before.

**Table 5:** Logit models for the probability of meeting each quality of care indicator

				GPs per 1000	Distance to practice	Sample size	ELSA wave
Disease	Name	Mean	SD	OR	OR		
Hypertension	hehbpb	0.866	0.341	0.677	0.982	7354	2
	hehbp	0.680	0.466	7.353*	0.892	1595	2
	hehbpa	0.423	0.494	1.363	0.753**	1565	2
High cholesterol	hecholb	0.784	0.411	1.579	0.959	1664	2
	hecholc	0.486	0.500	15.38**	0.816*	1647	2
IHD	hehrta	0.847	0.360	0.019	1.378	411	2
	hecgstp	0.694	0.461	2.909	0.935	1314	2
	hebetall	0.703	0.459	10.235	0.044**	116	2
Stroke	hehbpb1	0.417	0.494	16.14	0.395	263	2–4
Diabetes	hesuga	0.934	0.248	0.916	1.502	1649	2–4
	hewee	0.802	0.399	6.087	1.033	951	2–4
	heaceall	0.500	0.500	0.996	0.783	1950	2–4
	heftchk	0.831	0.375	28.25**	0.760	2014	2–4
	heslfcr	0.238	0.426	0.220	0.792	2015	2–4
	heslfcb	0.349	0.477	1.304	0.836	2015	2–4
	hechol	0.856	0.352	3.866	1.060	298	2
Osteoarthritis	hekneb	0.318	0.466	1.808	0.674*	1182	2–4
	heartall	0.176	0.381	11.09*	0.997	3768	2–4
	hearte	0.446	0.497	47.54	0.572*	804	2–4
	heartd	0.783	0.412	5.124	0.807	1398	2–4
	hepaf	0.387	0.488	0.236	1.083	278	2–4
Osteoporosis	heoste	0.540	0.499	0.752	1.084	581	2
	heosted	0.886	0.318	11.94	0.662	343	2
Prevention of falls	heflall	0.259	0.438	1.484	0.754	956	2 & 4
	hefld	0.346	0.476	4.100	0.281	968	2 & 4
Pain	hepai	0.771	0.421	4.992	0.943	462	2 & 4
Incontinence	heincall	0.223	0.417	16.70	0.623*	519	2
	heinctall	0.516	0.500	2.394	0.817	524	2
	heincth	0.619	0.486	0.345	0.950	524	2
	heinctg	0.715	0.452	21.02*	0.939	522	2
Hearing	hehrc	0.734	0.442	1.983	1.167	1547	2
	hehrall	0.902	0.298	124.50**	0.704*	771	2
Vision	hedreye	0.571	0.495	0.950	0.963	591	2
Depression	hepsye	0.471	0.500	0.811	1.038	447	2 & 4
	hepsyb	0.855	0.352	2.413	0.758	339	2 & 4

Controls are included in every model for demographics, marital status, ethnic group, self-reported health, education, employment status, household financial wealth, demographic profile of the area, percentage with no qualifications in area of residence and year

*OR* odds ratio, *GP* general practitioner

* *P* < 0.1, ** *P* < 0.05, *** *P* < 0.01

We find evidence of a significant effect of primary care supply for 11 of the 35 indicators, mainly involving activities that require additional GP/nurse time during the consultation rather than indicators related to referrals and prescription. The specific QoC indicators with a significant relationship with the supply of primary care services are the following:

- Has a doctor or nurse explained high blood pressure in a way you could understand? (Hypertension)
- Have doctors or nurses given you any choice about how to treat your high blood pressure? (Hypertension)
- Have doctors or nurses taken your preferences into account when making treatment decisions about your high cholesterol? (High cholesterol)
- Did any doctor ever tell you that you should take a medication called a betablocker? (IHD)
- In the past year, has any doctor or nurse examined your bare feet? (Diabetes)
- Doctor or nurse suggested physiotherapy for your knee pain? (Osteoarthritis)
- Doctor or nurse ever talked to you about how to keep your pain from getting worse? (Osteoarthritis)
- Did any doctor or nurse recommend you to try paracetamol before other medicines? (Osteoarthritis)
- Did a doctor or nurse ask you to provide a sample of urine for testing? (Incontinence)
- Did doctor or nurse take targeted history? (Incontinence)
- Did you get a hearing aid? And did a doctor teach you how to use your hearing aid? (Hearing)

The number of GPs per 1000 registered patients has a significant and positive impact on individual indicators of quality of care in the hypertension, high cholesterol, diabetes, osteoarthritis, hearing and incontinence disease areas. We found a negative and significant effect of distance to practice in hypertension, high cholesterol, IHD, arthritis, hearing and incontinence.

### Robustness checks

Our main finding was that QoC is positively correlated with primary care supply. We ran a series of analyses using alternative model specifications to check the robustness of our findings. Our preferred model is the 3-level model in Table 3, which was the starting point for our robustness checks (see Table 7 in Appendix).

First, we explored the potential non-linear effect of primary care supply on quality of care by including second- and third-order polynomial functions of the GP density and GP distance variables. The squared and cubic terms were non-significant for both indicators.

Second, we included a longer list of area characteristics, adding each of the Domains of the 2004 Index of Multiple Deprivation separately (i.e. income, employment, health, education, crime, and environment domain). The inclusion of these area indicators did not affect the sign, significance or order of magnitude of the main results. In addition, we ran an IV specification using this set of IMD variables as instruments. This model only accounted for the indicators nested within individuals’ structure and assumed a linear regression, as otherwise the models were computationally very challenging and not possible to run. We found a significant and positive effect on the number of GPs, although the effect of the GP distance variable remained negative but became non-significant. Note the limitations of this model, in that it does not account for the binary nature of the dependent variable and ignores clustering by individuals within PCTs.

Similar results were obtained when we attempted to control for secondary care supply using a range of measures from the CARAN report [42]. We included two variables which measure the average capacity of acute providers and the average distance to acute providers. We found that the inclusion of the distance to acute provider indicator had an impact on the effect of GP distance, which remained negative but became non-significant. The effect of the number of GPs per 1000 patients remained positive and strongly significant.

Finally, in the models that used more than 1 year of data we also explored the impact of accounting for potentially repeated values, and the impact of applying a different linkage for the GP density indicator. Some indicators are either not time limited or refer to whether or not a patient has ever achieved a quality of care indicator (e.g. Have you ever participated in a course or class about diabetes?). For the indicators included in more than one wave it is possible that the responses are simply repeated values. We explored the impact of this by dropping observations where a positive answer could have been repeated from previous years. The results in the individual indicator and disease domain models did not change appreciably and the outcomes were qualitatively similar in terms of the sign and statistical significance. Similarly, we found the same conclusions as reported in the paper when, in order to introduce more variation in the GP density variable, we used a different linkage by mapping data on the number of GPs from 2006, 2007 and 2009 to waves 2, 3 and 4, respectively (results for individuals indicator and disease domain models not shown since they do not apply to the base case model).

### Predicting PCT allocation of GP workforce to achieve QoC targets

We estimated that the increase in the GP workforce in England required for every PCT to achieve an optimal level of QoC, as defined by QOF targets of 70 and 90%, would be 17,278 and 69,551, respectively (Table 8 in Appendix). This is based on the marginal effect of the number of GPs on QoC estimated by the 3-level model using the information on the 35 indicators from wave 2. Given the current number of FTE GPs of 37,000 these increases would represent an increase in the size of the GP workforce by around 47% to achieve 70% QoC and 190% to achieve 90% QoC.

## Discussion

In this paper we explored the impact of primary care supply on quality of primary care provided in England to older adults. We found that individuals living in areas with a larger number of GPs per 1000 registered patients and in areas where the average distance to the general practices was shorter had a higher probability of achieving the minimum standards of care relevant for their conditions. Our findings were robust to alternative specifications. When analysing the impact for different subsets of QoC indicators, we found the impact to be concentrated in indicators of care related with CVD and arthritis. The analysis of each of the individual indicators shed light onto the impact on some specific standards of care related to other diseases such as diabetes, hearing problems and incontinence. Furthermore, the analyses show that most of the individual indicators more likely to be achieved in areas with better provision of primary care were related to particularly time-consuming activities, such as explaining the condition to the patient, taking patient’s preferences into account, providing a choice of treatment or teaching the patient how to use a device, as opposed to other indicators related to prescribing and referrals for test or specialist visits.

The findings of this study are especially relevant at a time when the role of GPs is evolving, with health services being transferred from hospitals to community settings and with an increasingly influential role of GPs in the commissioning of NHS activities [43]. As mentioned, CCGs replaced PCTs in April 2013. CCGs are clinically led statutory NHS bodies responsible for the planning and commissioning of health care services for their local area, and include GPs as leading members [44]. Therefore, GPs are now required to develop new skills and take on new responsibilities, which could restrict their contact time with patients, effectively reducing the supply of GPs.^5^, ^6^ While further investigation is warranted to evaluate the impact of CCGs on the care of the population its serves, our study may shed light on the potential negative impact of this on the quality of primary care services. Furthermore, while it is beyond the scope of this paper to evaluate whether the costs of expanding the primary care workforce would be considered cost-effective, we have estimated that the increase in GP supply required to achieve QoC targets are substantial. For every PCT to reach a 70% QoC target, a 47% increase in GP workforce would be required, and a 190% increase is required to meet the 90% target, holding everything constant.

A strength of our analysis is the richness of our data. We have information for a comprehensive list of individual indicators of quality of care which were meticulously derived. Previous work has focused on fewer indicators of quality of care and a narrower set of conditions, and has in some cases concluded that primary care supply has no effect on QoC, based on the lack of effect found on the indicators under study (e.g. [15]). With the analysis conducted in this paper we were able to identify the specific indicators and conditions where primary care supply has a significant impact on quality of care. However, we acknowledge that the small sample size for some specific indicators might imply a lack of statistical power to detect an effect in some measures. The multilevel approach that combined all available indicators allowed us to exploit multiple responses in an appropriate way and to take a system-wide perspective to investigate whether supply of primary care services has an impact on quality of care overall.

It is worth noting that national data on the quality of primary care have been reported annually as part of QOF since 2004 in the UK. We did not use QOF data in our analysis for two reasons. First, because QOF data are only available at the practice level, it is difficult to account for patient level factors — antecedent characteristics [45] — that may influence process measures. Second, because there is a risk of confounding due to the financial incentives that QOF provides [46, 47].

This study has a number of limitations. First, the QoC indicators are based on self-reported data. Previous work comparing quality measurement for the care of vulnerable elders by interview with examination of medical records has shown that self-reports tend to score the same or higher than medical records, which might suggest that they remember care which was not documented on their medical records, or they report receiving care when they have not [48]. However, as noted in Steel et al., 2008 [11], the data we use in this study shows high levels of agreement with similar indicators in the general practice contract which provide some level of validation. Second, quality of care indicators used in this study pertain to patients who have been diagnosed for a series of conditions, but we have no information on the number of undiagnosed individuals which might also be related to the provision of GPs, i.e. the greater the GP supply the more likely that patients are appropriately diagnosed and treated accordingly. Thirdly, our primary care supply variables were GPs per 1000 registered patients and distance to practice. These measures, especially GP density, have been used in previous studies. Alternative measures might include supply of other members of the primary care team, such as practice nurses, who are becoming increasingly important in primary care in England. This might be important if other primary care staff are employed as substitutes for GPs, in which case areas with lower supply of GPs might not have lower primary care supply overall. Previous work [49] has found that the number of GPs and the number of practice nurses are positively correlated, but further research using alternative primary care supply variables would be beneficial. Related to this issue, the aim of our study was to explore the relationship between the level of primary care supply and quality of care; a number of other practice level characteristics (e.g. mean GP age, single-handed practice, etc.) might also influence the level of quality of care offered; exploring the effect of these factors was beyond the scope of this paper. Finally, there might be a concern that our results are affected by endogeneity (i.e. reverse causality, omitted variables bias or measurement errors). A potential source of endogeneity is related to the fact that GPs might choose to work in areas where the quality of care is already below or above average. Potential mechanisms for this reverse relationship would be related to the type of area and the types of patients living in it, which are variables we control for in our models. Furthermore, the methods we use account for unobserved individual and area heterogeneity by exploiting the repeated responses of each individual across indicators and, when available, over time by a means of multilevel analyses. We attempted running area-level fixed-effect models among QoC indicators available in more than 1 year of data. However, due to the lack of variation of the GP supply variable across years these models were not possible. Similarly, we could not include individual or indicators fixed effects, as the GP supply variables did not vary within individuals. Nonetheless, in supplementary analysis we added a more comprehensive list of area-level characteristics and found no effect on the relationship observed in our models between primary care supply and quality of care. This is in line with previous research that has found no evidence of endogeneity between GP supply and quality of care [15].

## Appendix

See Tables 6, 7 and 8

**Table 6:** Definition of quality of care indicators

Disease	Name	Description
Hypertension	hehbpb	If aged 50 or older & remains hypertensive after nonpharmacological intervention:
		Did a doctor or nurse ever suggest that you take medication to lower your blood pressure?
	hehbp	If aged 50 or older and hypertensive:
		Has a doctor or nurse explained high blood pressure in a way you could understand?
	hehbpa	If aged 50 or older and hypertensive:
		Have doctors or nurses given you any choice about how to treat your high blood pressure?
High cholesterol	hecholb	If aged 50 or over and has high cholesterol:
		Has a doctor or nurse explained high cholesterol in a way you could understand?
	hecholc	If aged 50 or over and has high cholesterol:
		Have doctors or nurses taken your preferences into account when making treatment decisions about your high cholesterol?
IHD	hehrta	If aged 50 or older & has established CHD & is not on warfarin:
		Did a doctor suggest that you take medication to thin your blood?
	hecgstp	If aged 50 or older & has established CHD & smokes:
		Has a doctor or nurse ever advised you to stop smoking?
	hebetall	If aged 50 or older & has had a recent myocardial infarction:
		Did any doctor ever tell you that you should take a medication called a betablocker?
Stroke	hehbpb1	If aged 50 or older and has had a stroke: had a previous stroke
		Doctor or nurse ever suggested you take any medication to lower your blood pressure?
Diabetes	hesuga	If aged 50 or over and has diabetes:
		Glycosylated haemoglobin or fructosamine test performed in the past 12 months?
	hewee	If diabetic person aged 50 or older & not have established renal disease & not receiving an ACE inhibitor or angiotensin II receptor blocker:
		Urine test for protein in the last 12 months?
	heaceall	If diabetic person aged 50 or older & has one additional cardiac risk factor (i.e., smoker, hypertension, or renal insufficiency/microalbuminuria):
		Doctor discussed whether you should take ACE inhibitor or A2 receptor blocker?
	heftchk	If aged 50 or over and has diabetes:
		In the past year, has any doctor or nurse examined your bare feet?
	heslfcr	If aged 50 or over and has diabetes:
		Have you ever participated in a course or class about diabetes?
	heslfcb	If aged 50 or over and has diabetes:
		How much do you think you know about managing your diabetes?
	hechol	If aged 50 or over and has diabetes & has a fasting total cholesterol level of 5 mmol/l or greater:
		Has any doctor talked to you about how to lower your cholesterol?
Osteoarthritis	hekneb	If ambulatory person aged 50 or older & has had a diagnosis of symptomatic osteoarthritis of the knee for longer than 3 months & has no contraindications to exercise:
		Doctor or nurse suggested physiotherapy for your knee pain?
	heartall	If ambulatory person aged 50 or older & has had a diagnosis of osteoarthritis:
		Doctor or nurse ever talked to you about how to keep your pain from getting worse?
	hearte	If aged 50 or older & has had a diagnosis of osteoarthritis:
		Did any doctor or nurse recommend you to try paracetamol before other medicines?
	heartd	If aged 50 or older & has had a diagnosis of osteoarthritis:
		Has any doctor ever talked to you about what the specific purpose of the treatment is?
	hepaf	If aged 50 or older with severe symptomatic osteoarthritis of the knee or hip, has failed to respond to non- pharmacological and pharmacological therapy:
		Did any doctor recommend that you should have surgery or joint replacement?
Osteoporosis	heoste	If aged 50 or older & has untreated osteoporosis:
		Has any doctor or nurse recommended taking calcium pills or vitamin D?
	heosted	If woman aged 50 or older & is newly diagnosed with osteoporosis:
		Were these medicines recommended within 3 months?
Prevention of falls	hefld	If aged 65 or older & reported 2 or more falls in the past year, or a single fall with injury requiring treatment:
		With any of your past falls, did a doctor or nurse to try to understand why you fell?
	heflall	If aged 65 or older & reported 2 or more falls in the past year, or a single fall with injury requiring treatment:
		Did a doctor recommend any additional tests to understand why you fell?
Pain	hepai	If aged 50 or older & has a newly reported chronic painful condition:
		Did your doctor or nurse recommend any treatments for your pain?
Incontinence	heincall	If aged 65 or older & has new UI that persists for over 1 month:
		Doctor or nurse took targeted history?
	heinctall	If aged 65 or older & has new UI that persists for over 1 month:
		Did a doctor or specialist such as an urologist or gynaecologist perform an internal exam?
	heincth	If aged 65 or older & has new UI that persists for over 1 month:
		Did a doctor or nurse talk with you about how to treat urinary incontinence?
	heinctg	If aged 65 or older & has new UI that persists for over 1 month:
		Did a doctor or nurse ask you to provide a sample of urine for testing?
Hearing	hehrc	If aged 65 or older & has a problem with hearing:
		Did doctor refer you to an ear specialist to check your hearing?
	hehrall	If aged 65 or older & is a hearing aid candidate:
		Did you get a hearing aid? And did a doctor teach you how to use your hearing aid?
Vision	hedreye	If aged 50 or older & is diagnosed with a cataract that limits the patient’s ability to carry out needed or desired activities:
		Did any doctor or optician recommend that you have your cataracts removed?
Depression	hepsye	If aged 50 or older & receives a diagnosis of a new depression episode:
		When you talked about these feelings, did doctor ask if you had thoughts about suicide?
	hepsyb	If aged 50 or older is diagnosed with clinical depression: diagnosed with clinical depression
		Did you start medication or counselling within 2 weeks of being offered this treatment?

**Table 7:** Robustness checks

	OR	ME
Base case model		
GPs per 1000 patients	2.453**	0.203***
Distance to practice (km)	0.918**	−0.020**
Quadratic functional form		
GPs per 1000 patients	5.957	
GPs per 1000 patients squared	0.535	
Distance to practice (km)	0.779*	
Distance to practice (km) squared	1.030	
Cubic functional form		
GPs per 1000 patients	0.400	
GPs per 1000 patients squared	33.953	
GPs per 1000 patients cubic	0.130	
Distance to practice (km)	0.918	
Distance to practice (km) squared	0.963	
Distance to practice (km) cubic	1.007	
Adding IMD 2004 variables		
GPs per 1000 patients	2.982***	
Distance to practice (km)	0.925**	
Adding secondary care indicators		
GPs per 1000 patients	2.699***	
Distance to practice (km)	0.953	
Using IMD 2004 variables as instruments		
GPs per 1000 patients	N/A	0.373***
Distance to practice (km)	N/A	−0.024
Adding IMD 2004 and secondary care indicators		
GPs per 1000 patients	3.155***	
Distance to practice (km)	0.946	

*OR* odds ratio, *ME* marginal effect, *GP* general practitioner

* *P* < 0.1, ** *P* < 0.05, *** *P* < 0.01. Controls are included in every model for demographics, marital status, ethnic group, self-reported health, education, employment status, household financial wealth, demographic profile of the area and percentage with no qualifications in area of residence

**Table 8:** PCTs distribution of GP workforce to meet QoC targets

PCT	Mean QoC	GPs (per 1000 patients)	Total number of GPs	Total number of registered patients	ΔGPs required to achieve 70% target^a^	ΔGPs required to achieve 90% target^a^
1	0.538	0.480	119	248,158	197.90	442.12
2	0.691	0.606	110	181,641	7.72	186.48
3	0.600	0.526	169	321,435	158.17	474.50
4	0.670	0.422	111	262,986	38.82	297.63
5	0.591	0.555	207	372,760	200.10	566.94
6	0.704	0.529	141	266,460	−5.35	256.88
7	0.602	0.540	153	283,473	136.64	415.61
8	0.604	0.408	72	176,377	83.17	256.75
9	0.639	0.593	160	269,845	81.14	346.70
10	0.646	0.595	137	230,348	60.82	287.51
11	0.591	0.530	170	320,594	172.09	487.60
12	0.592	0.514	144	280,198	149.53	425.28
13	0.519	0.475	77	162,197	144.84	304.46
14	0.582	0.675	120	177,699	103.10	277.98
15	0.714	0.586	141	240,554	−16.91	219.83
16	0.634	0.569	156	274,063	88.81	358.52
17	0.572	0.558	117	209,784	131.65	338.11
18	0.759	0.592	55	92,890	−26.93	64.48
19	0.611	0.602	114	189,375	82.83	269.20
20	0.661	0.579	95	164,027	31.84	193.26
21	0.635	0.501	158	315,374	100.41	410.78
22	0.676	0.542	59	108,814	12.75	119.83
23	0.711	0.713	188	263,789	−14.72	244.88
24	0.647	0.585	137	234,372	61.05	291.71
25	0.794	0.537	158	294,130	−135.78	153.68
26	0.582	0.511	104	203,695	118.45	318.92
27	0.745	0.566	109	192,507	−43.06	146.39
28	0.679	0.482	98	203,339	20.77	220.88
29	0.617	0.502	96	191,059	78.01	266.04
30	0.622	0.563	143	253,938	97.60	347.51
31	0.598	0.548	170	310,477	156.29	461.83
32	0.655	0.542	82	151,159	33.34	182.10
33	0.620	0.523	149	284,628	112.65	392.76
34	0.594	0.461	169	366,640	191.13	551.95
35	0.578	0.434	112	257,835	154.38	408.13
36	0.672	0.578	116	200,603	27.76	225.18
37	0.644	0.505	78	154,493	42.23	194.27
38	0.631	0.450	105	233,546	79.56	309.40
39	0.655	0.497	102	205,280	45.28	247.30
40	0.663	0.678	70	103,249	19.05	120.66
41	0.606	0.543	130	239,218	110.20	345.62
42	0.629	0.592	113	190,944	66.83	254.75
43	0.596	0.608	123	202,448	103.86	303.09
44	0.546	0.508	176	346,204	261.68	602.39
45	0.571	0.544	125	229,703	145.32	371.38
46	0.614	0.662	156	235,513	100.08	331.86
47	0.635	0.636	140	220,021	70.79	287.32
48	0.608	0.580	211	363,647	164.03	521.90
49	0.662	0.598	122	203,990	38.24	238.99
50	0.488	0.598	93	155,440	162.45	315.43
51	0.668	0.582	166	285,196	44.84	325.51
52	0.824	0.593	90	151,670	−92.19	57.07
53	0.500	0.566	142	251,069	247.08	494.17
54	0.555	0.464	128	276,098	197.51	469.22
55	1.000	0.579	106	183,005	−270.15	−90.05
56	0.604	0.576	141	244,963	115.99	357.06
57	0.722	0.599	212	353,784	−38.69	309.48
58	0.565	0.607	177	291,564	194.38	481.31
59	0.714	0.598	171	285,786	−20.09	261.16
60	0.518	0.605	197	325,864	292.06	612.75
61	0.652	0.546	128	234,557	55.20	286.03
62	0.737	0.565	164	290,282	−53.38	232.29
63	0.601	0.593	224	377,828	183.94	555.77
64	0.642	0.606	177	291,951	83.17	370.49
65	0.565	0.489	130	265,898	176.35	438.02
66	0.600	0.602	116	192,597	94.77	284.31
67	0.725	0.600	240	400,117	−48.96	344.80
68	0.658	0.522	105	200,982	41.21	239.00
69	0.608	0.552	191	345,922	156.87	497.30
70	0.569	0.538	90	167,149	107.90	272.40
71	0.534	0.457	118	258,188	211.27	465.35
72	0.585	0.502	157	312,779	177.14	484.95
73	0.672	0.568	442	778,374	107.72	873.74
74	0.683	0.539	220	408,538	34.32	436.37
75	0.715	0.591	204	345,169	−25.40	314.29
76	0.609	0.683	372	544,587	245.05	780.99
77	0.518	0.556	169	304,073	272.21	571.45
78	0.594	0.611	425	695,547	361.12	1045.62
79	0.689	0.535	151	282,017	15.71	293.25
80	0.665	0.538	349	648,411	112.78	750.90
81	0.581	0.545	381	699,007	408.41	1096.32
82	0.575	0.465	119	255,889	157.39	409.22
83	0.569	0.535	141	263,485	170.33	429.63
84	0.645	0.661	341	515,497	139.10	646.42
85	0.627	0.651	335	514,480	184.41	690.72
86	0.672	0.566	189	334,124	45.82	374.64
87	0.642	0.550	251	456,692	130.65	580.09
88	0.591	0.539	206	382,357	205.25	581.53
89	0.633	0.559	156	279,143	91.80	366.51
90	0.731	0.639	214	334,655	−51.27	278.08
91	0.703	0.630	299	474,435	−8.14	458.76
92	0.728	0.514	163	316,851	−44.16	267.66
93	0.681	0.635	163	256,697	24.19	276.81
94	0.611	0.592	273	461,315	202.67	656.67
95	0.576	0.560	122	217,730	132.56	346.83
96	0.706	0.511	115	225,209	−6.52	215.12
97	0.648	0.546	281	514,934	131.76	638.52
98	0.616	0.643	503	781,790	323.63	1093.01
99	0.609	0.610	189	309,641	139.11	443.84
100	0.569	0.547	155	283,286	182.28	461.07
101	0.560	0.619	325	524,846	362.05	878.57
102	0.531	0.572	199	348,056	288.64	631.17
103	0.728	0.604	250	414,061	−56.66	350.83
104	0.639	0.545	311	570,717	171.62	733.27
105	0.571	0.596	340	570,343	360.83	922.12
106	0.589	0.579	648	1119,201	610.09	1711.52
107	0.651	0.642	514	801,236	193.26	981.78
108	0.748	0.607	206	339,480	−79.76	254.33
109	0.672	0.583	103	176,659	24.11	197.96
110	0.589	0.528	360	682,310	371.18	1042.66
111	0.613	0.565	372	658,609	282.26	930.41
112	0.705	0.449	154	342,899	−7.67	329.79
113	0.617	0.528	357	676,316	276.14	941.72
114	0.673	0.577	176	305,151	40.43	340.73
115	0.684	0.533	173	324,708	25.23	344.78
116	0.745	0.521	223	427,784	−94.93	326.06
117	0.703	0.569	115	202,108	−2.69	196.21
118	0.682	0.534	141	264,231	23.64	283.68
119	0.624	0.552	332	601,251	224.95	816.65
120	0.614	0.623	352	565,182	238.20	794.41
121	0.582	0.589	313	531,164	309.48	832.21
122	0.573	0.558	91	163,070	101.64	262.12
123	0.638	0.660	386	585,077	178.11	753.90
124	0.642	0.677	495	731,525	208.40	928.31
125	0.576	0.594	132	222,042	135.39	353.90
126	0.563	0.603	359	595,114	402.65	988.31
127	0.683	0.594	163	274,187	23.35	293.19
128	0.570	0.558	173	309,921	197.69	502.69
129	0.646	0.583	214	366,931	96.80	457.90
130	0.616	0.551	221	401,313	166.34	561.28
131	0.543	0.549	406	739,083	572.59	1299.94
132	0.617	0.562	711	1,265,468	514.03	1759.41
133	0.731	0.605	313	517,634	−79.19	430.22
134	0.710	0.574	376	655,623	−32.26	612.95
135	0.705	0.535	259	483,819	−12.39	463.75
136	0.588	0.498	201	403,390	222.43	619.42
137	0.593	0.607	362	596,632	313.30	900.46
138	0.731	0.589	264	448,485	−67.90	373.46
139	0.545	0.681	304	446,158	341.16	780.23
140	0.668	0.740	378	510,936	81.13	583.95
141	0.687	0.711	281	394,974	25.57	414.28
142	0.709	0.620	216	348,161	−15.80	326.84
143	0.655	0.731	391	535,079	117.77	644.35
144	0.672	0.810	600	740,899	100.41	829.55
145	0.656	0.622	85	136,552	29.74	164.12
146	0.718	0.563	78	138,436	−12.02	124.22
147	0.656	0.909	290	319,067	69.78	383.78
148	0.500	0.407	91	223,474	219.93	439.85
149	0.792	0.699	101	144,533	−65.75	76.49
150	0.602	0.614	129	210,008	100.99	307.66
152	0.664	0.597	149	249,644	44.57	290.25
Total estimated ΔGPs required to achieve QoC target					17,278	69,551

*PCT* Primary Care Trusts, *QoC*  quality of care, *GP* general practitioners

^a^Based on estimated marginal effect (ME) of GP per 1000 patients on QoC of 0.203 and applying the following formulae = (Target QoC − QoC)/ME)*Registered patients/1000

## Footnotes

1
http://webarchive.nationalarchives.gov.uk/20080814090357/ic.nhs.uk/statistics-and-data-collections/population-and-geography/pct-mapping-tool.

2
http://www.hscic.gov.uk/workforce.

3
http://collections.europarchive.org/tna/20100509080731/ic.nhs.uk/statistics-and-data-collections/population-and-geography/pct-mapping-tool.

4
http://www.neighbourhood.statistics.gov.uk/.

5
http://www.gponline.com/News/article/1110486/huge-variation-money-paid-gp-commissioners/.

6
http://www.guardian.co.uk/society/datablog/2012/mar/12/gp-commissioning-data-false-economy.
